# ITS Polymorphisms Shed Light on Hybrid Evolution in Apomictic Plants: A Case Study on the *Ranunculus auricomus* Complex

**DOI:** 10.1371/journal.pone.0103003

**Published:** 2014-07-25

**Authors:** Ladislav Hodač, Armin Patrick Scheben, Diego Hojsgaard, Ovidiu Paun, Elvira Hörandl

**Affiliations:** 1 Department of Systematics, Biodiversity and Evolution of Plants (with Herbarium), Georg August University Göttingen, Göttingen, Germany; 2 Division of Systematic and Evolutionary Botany, University of Vienna, Vienna, Austria; Royal Botanic Gardens, Kew, United Kingdom

## Abstract

The reconstruction of reticulate evolutionary histories in plants is still a major methodological challenge. Sequences of the ITS nrDNA are a popular marker to analyze hybrid relationships, but variation of this multicopy spacer region is affected by concerted evolution, high intraindividual polymorphism, and shifts in mode of reproduction. The relevance of changes in secondary structure is still under dispute. We aim to shed light on the extent of polymorphism within and between sexual species and their putative natural as well as synthetic hybrid derivatives in the *Ranunculus auricomus* complex to test morphology-based hypotheses of hybrid origin and parentage of taxa. We employed direct sequencing of ITS nrDNA from 68 individuals representing three sexuals, their synthetic hybrids and one sympatric natural apomict, as well as cloning of ITS copies in four representative individuals, RNA secondary structure analysis, and landmark geometric morphometric analysis on leaves. Phylogenetic network analyses indicate additivity of parental ITS variants in both synthetic and natural hybrids. The triploid synthetic hybrids are genetically much closer to their maternal progenitors, probably due to ploidy dosage effects, although exhibiting a paternal-like leaf morphology. The natural hybrids are genetically and morphologically closer to the putative paternal progenitor species. Secondary structures of ITS1-5.8S-ITS2 were rather conserved in all taxa. The observed similarities in ITS polymorphisms suggest that the natural apomict *R. variabilis* is an ancient hybrid of the diploid sexual species *R. notabilis* and the sexual species *R. cassubicifolius*. The additivity pattern shared by *R. variabilis* and the synthetic hybrids supports an evolutionary and biogeographical scenario that *R. variabilis* originated from ancient hybridization. Concerted evolution of ITS copies in *R. variabilis* is incomplete, probably due to a shift to asexual reproduction. Under the condition of comprehensive inter- and intraspecific sampling, ITS polymorphisms are powerful for elucidating reticulate evolutionary histories.

## Introduction

Hybridization and polyploidy are creative evolutionary forces in plant radiations, and most or perhaps all angiosperms are either polyploid or of ancient polyploid origin [1]. High morphological plasticity and large ecological amplitude of allopolyploids could be derived from an increased propensity for epigenetic variation following interspecific hybridization [2]. Rieseberg and collaborators [3] proposed that ancestral phenotypic and genomic traits of recent hybrids might be recognized by comparison with experimental hybrids. Hybrid speciation is facilitated in plants through subsequent polyploidization [4] and can be connected to a transition from sexuality to apomixis, i.e., asexual reproduction via seed [5]. Nonetheless, the reconstruction of hybrid relationships and parentage in natural systems is still a methodological challenge.

The internal transcribed spacer (ITS) of the nrDNA has been established as a standard molecular marker to infer generic and interspecific relationships in flowering plants [6]–[8] and to infer hybridization events in other eukaryotes including algae, heterotrophic protists, invertebrates and vertebrates [9]–[15]. ITS nrDNA has proved to be a helpful non-coding marker to infer hybridization events because of its biparental inheritance and its occurrence in hundreds to thousands of copies within a single genome [8], [16], [17]. The majority of systematics studies aim at reconstruction of phylogenetic relationships using ITS in combination with maternally inherited plastid marker (e.g., [18]–[23]).

According to Song and collaborators [24], a genome of each plant individual contains 35 ITS variants on average. The homogenization of different progenitor ITS variants in sexual hybrids through concerted evolution is a well-documented phenomenon [25]–[27]. In apomictic plant lineages, the homogenizing effects of meiosis are lacking, and thus heterozygous ITS polymorphisms inherited from hybrid ancestry may persist over several generations [21], [28]–[30]. Only few studies have analyzed ITS polymorphisms in apomictic hybrids [21], [28], [29], [31], [32]. The features of secondary structure of ITS in apomictic plants, however, are still unknown.

Apart from the lasting popularity of this marker, there is still a lively debate whether a direct link exists between ITS structural features like compensatory base changes (CBCs) and Ernst Mayr's biological species concept [33]–[35]. CBCs are mutations that occur in a primary RNA transcript, whereby both nucleotides paired in the secondary configuration of the ITS transcript mutate so that their bond is retained (e.g., G-C mutates to A-U). A hemi-CBC (hCBC) is the mutation of one of the two nucleotides while maintaining the nucleotide bond. Non-compensatory base changes alter the secondary structure by disabling pairing within any of the helical regions (i.e., within the internal paired regions). According to [34], the occurrence of CBCs in ITS2 secondary structure between two sexual species is correlated to unsuccessful interspecific mating. This assumption is based solely on coincidence, i.e., similar rates of evolutionary changes affecting both the ITS sequences and the genes involved in sexual reproduction [33], [36]. Hence, the appearance of a CBC between species could theoretically serve as a criterion for delimitation of sexual species. Reflecting the difficulty associated with multiple copies of the ITS within a genome, analysis of an extensive ITS2 dataset obtained by high-throughput sequencing in plants suggests that intragenomic CBCs are unlikely. Thus, CBCs can potentially serve as species-specific markers [24], [34].

Despite the wide usage of the ITS region, only a few studies have discussed obvious difficulties regarding the presence of both functional and putative non-functional copies (e.g., [37]–[41]). The homogenization of rRNA genes through gene conversion prevents these from accumulating mutations [42]. The most frequently employed criteria to recognize putative non-functional ITS copies (also referred to as “pseudogenes”) are nucleotide substitutions in highly conserved motifs (especially within 5.8S sequence regions), occurrence of methylation-induced substitutions (C → T, G → A), decreased or varying GC content, incorrect secondary structure folding and incongruent phylogenetic placement of conspecific ribotypes [21], [37]. For this reason, frequent occurrence of putative non-functional copies may be a reliable hint for the incompleteness or even suppression of concerted evolution of the ITS1-5.8S-ITS2 copies [21], [39], .

**Figure 1:**
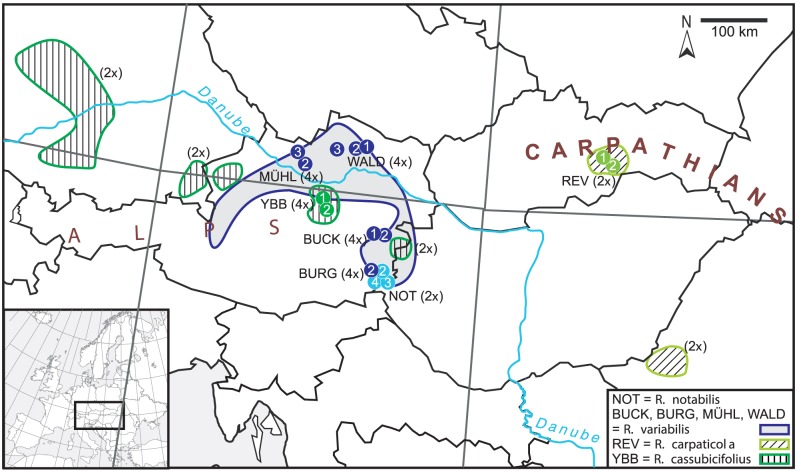
Distribution of the analyzed populations of the *Ranunculus auricomus* complex. Dots represent sampled populations, the areas with the same color show the entire distribution of the respective taxon within Central Europe (after [49]).

As in many other plant genera [45], hybridization and polyploidization have shaped the evolutionary history of buttercups (*Ranunculus*) [29], [46]–[51]. The Euro-Siberian *R. auricomus* complex, which belongs to one clade classified as *R*. sect. *Auricomus* [51], comprises about 800 polyploid apomictic microspecies and only four sexual species [52]. Here we investigate three sexual species (*R*. *carpaticola* Soó, *R*. *cassubicifolius* W. Koch, *R*. *notabilis* Hörandl and Guterm.) and one apomictic species (*R*. *variabilis* Hörandl and Guterm.) from Central Europe. These species are taxonomically subdivided into two morphological collective groups: the *auricomus* group ( = *R*. *notabilis*, *R*. *variabilis*) and the *cassubicus* group ( = *R*. *carpaticola*, *R*. *cassubicifolius*) [53]. According to population genetic distances, these two groups separated about 0.9 Ma ago [54]. Autotetraploid *R. cassubicifolius* and diploid *R. carpaticola* are woodland species of the pre-Alps and Carpathians, respectively, whereas diploid *R. notabilis* is endemic in a small lowland area in southeastern Austria and occurs in woods and meadows; allotetraploid *R. variabilis* is widespread in lowlands of Austria, sympatric with *R. notabilis* and *R. cassubicifolius*, but colonizing mostly anthropogenic meadows (Figure 1). Allozyme studies and morphometrics based on leaves and fruit characters suggested that diploid *R. notabilis* (*auricomus* lowland group) is a putative progenitor of tetraploid *R. variabilis* [55]. Surprisingly, experimental crosses between 4*x R. cassubicifolius* and 2*x R. notabilis*, employing the latter as pollen donor, resulted in triploid synthetic hybrids that resembled *R. variabilis* in basal leaf shape. Since the geographical range of *R. variabilis* spans over that of the sexual species (Figure 1), we hypothesize an allopolyploid, Pleistocene origin of *R. variabilis* from hybrids of *R. cassubicifolius* and *R. notabilis*. The fourth known sexual species of the *R. auricomus* complex (i.e., *R. marsicus* Guss. et Ten.) was not taken into account because its distribution area is restricted to the Central Apennines in Italy, which is geographically isolated from all other species under study [56]. Moreover, it is tetraploid and hexaploid and therefore must have another evolutionary origin. All sexual species of the complex belong to one clade but the internal relationships of this clade are not yet well resolved [57]. However, the other species of this clade (i.e., of section *Auricomus*) occur in other continents, and therefore interspecific hybridization is unlikely.

Here we aim to shed light on intraspecific ITS nrDNA variability in closely related, but morphologically diversified taxa of the *Ranunculus auricomus* complex in Central Europe. Therefore, an extended sampling of ITS ribotypes on the intraspecific level will enable us to recover additivity patterns in hybrid genomes to better understand complicated reticulate relationships among sexuals and apomicts. We want to address the following specific questions: (1) Do ITS polymorphisms characterize the sexual species and the hybrids? (2) Are ITS polymorphisms observed in artificial hybrids similar to those in natural tetraploid apomictic *R. variabilis*, indicating allopolyploid origin? (3) Are the ITS data congruent with morphometric data in the putative parent-hybrid relationships? (4) Is secondary structure of the entire ITS1-5.8S-ITS2 region informative about species differentiation, concerted evolution and formation of putative non-functional copies?

## Materials and Methods

### Ethics statement

The collections used for this study did not involve endangered or protected species and no specific permissions were required for sampling activities in these locations.

### Plant material

DNA samples were collected from *R. carpaticola*, *R. cassubicifolius*, *R. notabilis*, and *R. variabilis* (Table 1). Additionally, homoploid and heteroploid experimental hybrids (F_1_ offspring) obtained from hand-made crosses of *R. carpaticola* × *notabilis* (11 individuals), as well as *R. cassubicifolius* × *notabilis* (14 individuals) were studied. In all crosses, *R. notabilis* was used as pollen donor; homoploid and heteroploid crosses were performed to reconstruct pathways to apomixis [58]. Ploidy levels of experimentally produced hybrids were assessed in mitotic cell divisions on root tips treated with α-bromonaphthalene during 6 h, 3 h at RT and 3 h at 5°C, and fixed 24–48 h in 3∶1 (ethanol∶acetic acid). Then, root tips were hydrolyzed 10 min in 1N HCl at 60°C and stained with Schiff's reagent (C_20_H_21_N_3_SO_3_). On average, five metaphase cells per plant were examined with a Leica B5500 microscope (Leica, Wetzlar, Germany). For provenance, accession codes, ploidy and method of reproduction of the sampled taxa see Table 1 and Figure 1. The DNA sequencing was performed on 68 individuals. Plants were cultivated at the Botanical Garden of the University of Göttingen, Germany. Herbarium material was provided by the herbarium of the University of Vienna, Austria (WU). Four individuals were selected for cloning and 19–20 clones per individual were sequenced, 79 in total. A triploid individual (G11) was selected rather than a diploid one because the maternal species from this cross occurs in the same geographical region as the putative natural hybrid (Figure 1). Cloned *R. cassubicifolius* 8472-1 represents the population used for crossing experiments. Another cloned individual *R. notabilis* 7223-10 was taken from an isolated population to minimize presumed secondary introgressions from *R. variabilis* [55]. Additionally, we cloned the individual *R. variabilis* 8210-5 belonging to a wild population geographically localized between those sexual species described above.

**Table 1:** List of directly sequenced individuals.

taxon	herbarium collection No. or garden accession No.	population	location/GPS coordinates	ploidy level	sexual or apomictic	GenBank accession No.
*R. notabilis*	7223-6/-10	NOT2	AT, Strem/47° 2' 58.0" N; 16° 26' 3.7" E	2*x*	sexa)	KF671993-KF671994
*R. notabilis*	5613-1/-2	NOT3	AT, Strem/47° 2' 58.0" N; 16° 26' 3.7" E	2*x*	sexa)	KF671996-KF671997
*R. notabilis*	7224-15	NOT3	AT, Strem/47° 0' 56.0" N; 16° 26' 38.5" E	2*x*	sexa)	KF671995
*R. notabilis*	9609-2/-5/-9	NOT3	AT, Strem/47° 2' 58.0" N; 16° 26' 3.7" E	2*x*	sexa)	KF671999-KF672001
*R. notabilis*	5615	NOT4	AT, Strem/47° 2' 58.0" N; 16° 26' 3.7" E	2*x*	sexa)	KF671998
*R. variabilis*	7221-13/-6/-9	BURG2	AT, Strem/47° 2' 51.3" N; 16° 25' 55.0" E	4*x*	apoa)	KF672008-KF672010
*R. variabilis*	8210-5/-7/-22/-23/-25	BUCK1	AT, Spratzau/47° 35' 19.5" N; 16° 12' 13.8" E	4*x*	apoa)	KF672002-KF672006
*R. variabilis*	8212-23	BUCK2	AT, Spratzau/47° 33' 25.3" N; 16° 18' 45.2" E	4*x*	apoa)	KF672007
*R. variabilis*	8214-1/-2	WALD1	AT, Rosenburg/48° 37' 52.0" N; 15° 38' 7.3" E	4*x*	apoa)	KF672017-KF672018
*R. variabilis*	8215-13	WALD2	AT, Wegscheid/48° 36' 25.1" N; 15° 28' 59.9" E	4*x*	apoa)	KF672011
*R. variabilis*	8216-26/-33	WALD3	AT, Zwettl/48° 35' 42.7" N; 15° 10' 2.6" E	4*x*	apoa)	KF672015-KF672016
*R. variabilis*	8218-24	MÜHL2	AT, Kefermarkt/48° 26' 31.6" N; 14° 33' 6.8" E	4*x*	apoa)	KF672012
*R. variabilis*	8222-22/-25	MÜHL3	AT, Reichenau/48° 27' 25.7" N; 14° 21' 24.2" E	4*x*	apoa)	KF672013-KF672014
*R. cassubicifolius*	8472-1/-3/-10/-19	YBB1	AT, Schwarzois/47° 54' 48.0" N; 14° 56' 37.4" E	4*x*	sexb)	KF672026-KF672029
*R. cassubicifolius*	8473-24/-25	YBB2	AT, Schwarzois/47° 52' 29.5" N; 14° 55' 59.7" E	4*x*	sexb)	KF672030-KF672031
*R. cassubicifolius*	A7, 17, 25, 35	8473×8473	DE, Göttingen, experimental crossing	4*x*	sexc)	KF672032-KF672035
*R. carpaticola*	8483-1/-4/-5/-6/-10	REV1	SK, Revúca/48° 41' 30.9" N; 20° 7' 34.9" E	2*x*	sexb)	KF672019-KF672025
*R. carpaticola*	8486-2/-4	REV2	SK, Revúca/48° 41' 21.9" N; 20° 5' 45.4" E	2*x*	sexb)	KF672023-KF672024
*R. carpaticola* × *notabilis*	F2, 4, 7, 10, 90	8483×7224	DE, Göttingen, experimental crossing	2*x*	sexc)	KF671968-KF671972
*R. carpaticola* × *notabilis*	J10, 20, 22, 25, 33, 35	8483×7224	DE, Göttingen, experimental crossing	2*x*	sexc)	KF671987-KF671992
*R. cassubicifolius* × *notabilis*	G1, 2, 5, 7, 9, 11, 15-20	8472×7220	DE, Göttingen, experimental crossing	3*x*	sexd)	KF671973-KF671984
*R. cassubicifolius* × *notabilis*	H4, 5	8473×7223	DE, Göttingen, experimental crossing	3*x*	sexd)	KF671985-KF671986

a) [96].

b) [46].

c) functional sexual seed, but with aposporous initials (for details see [58]).

d) functional sexual seed, but with low rates of aposporous seeds (for details see [58]).

### DNA extraction, PCR amplification, cloning and sequencing

Genomic DNA was extracted from silica-dried, herbarium or frozen (−80°C) plant material (stem leaves) using Tissue-Lyser II Qiagen and Invisorb Spin Plant Minikit (Invitek) following the manufacturer's instructions. PCR of the ITS1-5.8S-ITS2 region was performed according to [59] using primers 18sF and 26sR (after [60]); the PCR amplicons were cleaned with the Invisorb Spin PCRapid Kit (Invitek, Berlin, Germany). PCR products of a subset of four individuals (*Ranunculus notabilis* 7223-10, *R. cassubicifolius* 8472-1, *R. cassubicifolius* × *notabilis* G11, *R. variabilis* 8210-5; Table 2) were cloned using the TOPO TA cloning kit (Invitrogen, Carlsbad, CA, USA) and the pCR2.1-TOPO vector. Ligation products were integrated into competent cells of *Escherichia coli* TOP 10, as supplied by the manufacturer. Plasmid DNA was extracted and purified with a NucleoSpin-Plasmid kit (Macherey and Nagel, Düren, Germany) following the manufacturer's instructions. Sequencing reactions were performed with a Dye Terminator Cycle Sequencing v3.1 kit (Applied Biosystems, Darmstadt, Germany) and an ABI Prism 3100 (Applied Biosystems) automated sequencer. PCR primers were used for sequencing. Sequences were processed using the sequence analysis program Geneious Pro version 5.6.4 [61] and deposited in GenBank (KF671968- KF672114).

**Table 2:** List of cloned individuals.

specimen ( = clone library)	clone identifiers	population	location/GPS coordinates	ploidy level	sexual or apomictic	GenBank accession No.
*R. notabilis* 7223-10	N01-N20	NOT2	AT, Strem/47° 2' 58.0" N; 16° 26' 3.7" E	2*x*	sexa)	KF672076-KF672095
*R. variabilis* 8210-5	V01-V19	BUCK1	AT, Spratzau/47° 35' 19.5" N; 16° 12' 13.8" E	4*x*	apoa)	KF672096-KF672114
*R. cassubicifolius* 8472-1	S01-S20	YBB1	AT, Schwarzois/47° 54' 48.0" N; 14° 56' 37.4" E	4*x*	sexb)	KF672036-KF672055
*R. cassubicifolius* × *notabilis* G11	X01-X20	8472×7220	DE, Göttingen, experimental crossing	3*x*	sexc)	KF672056-KF672075

a) [96].

b) [46].

c) functional sexual seed, but with low rates of aposporous seeds (for details see [58]).

### Sequence alignment and data analysis

Sequences comprising ITS1-5.8S-ITS2 were aligned with Clustal W [62] and further adjusted manually in BioEdit 7.0.9.0 [63]. The online ITS2 database (http://its2.bioapps.biozentrum.uni-wuerzburg.de/) was used for annotation of the ITS2 region, whereas the ITS1 and 5.8S were annotated by comparison to published secondary structures (e.g., [64], [65]). The 5.8S region of directly sequenced accessions was completely conserved across the entire dataset, hence we excluded it from subsequent analyses and the final alignment consisted of ITS1+ITS2 regions (501 bp in total). The polymorphic sites were manually determined in BioEdit 7.0.9.0 [63] and visualized separately for ITS1 and ITS2 using an online application to create DNA weblogos (http://weblogo.berkeley.edu/). Polymorphic positions of ITS1, 5.8S and ITS2 were annotated using a single secondary structure model of *R. notabilis* (voucher specimen 5613-1) as uniform template (Figure S1). In order to visualize the reticulate relationships among the studied species, NeighborNet analysis was employed in SplitsTree4 4.10 [66], applying uncorrected P distances and ambiguities handled as average. Bootstrap support values for internal splits were calculated with 1000 replicates. Minimum energy models of the secondary structures for the ITS1-5.8S-ITS2 rRNA transcripts were obtained using RNAstructure 5.3 [67]. For comparison, the 5.8S secondary structures from all cloned ITS nrDNA sequences were homologically modeled based on the *R. notabilis* 5613-1 template using the online ITS2 database (http://its2.bioapps.biozentrum.uni-wuerzburg.de/) and subsequently aligned in 4SALE 1.7 [68]. The final graphical output was generated by Varna 3.8 [69] and edited in CorelDraw Graphics Suite X3. The GC contents within ITS1-5.8S-ITS2 regions were computed using MEGA 5.1. [70] and the resulting percentages for each clone were plotted as a 3D scatter plot in PAST 2.17c [71]. The cloned sequences were checked for putative non-functional ITS copies, chimeras and recombinants. First, the presence of angiosperm conserved motifs was determined as proposed by [40] in BioEdit 7.0.9.0 [63]. We focused especially on the nucleotide polymorphisms in the 5.8S region. This sequence region is strictly conserved among all directly sequenced individuals and the single nucleotide polymorphisms observed in clones may be a hint for putative PCR artifacts or non-functional ITS copies. The alignment containing ITS1-ITS2 was checked for recombinants and chimeras using the program RDP 4.17 [72]. No chimeric sequences or recombinants were detected. The DnaSP 5.10 software [73] was then used to delimitate ITS1-ITS2 ribotypes. Ribotype networks were constructed by statistical parsimony (with a parsimony probability set to 95%) in TCS 1.21 [74]. Finally, average Kimura 2-parameter genetic distances within and among groups were computed in MEGA 5.1. [70].

### Landmark geometric morphometrics

In order to quantify the phenotypic variation and test for possible overlaps among the four genetically different taxa of the *auricomus*-morphotype (*R. notabilis*, *R. carpaticola* × *notabilis*, *R. cassubicifolius* × *notabilis*, *R. variabilis*) we employed 2D-landmark geometric morphometric analyses. The *auricomus* morphotype shows a heterophyllous sequence of basal leaves with deeply divided leaf blades formed during the flowering period (basal leaves 3, 4, and 5 sensu [75], whereas in the *cassubicus* morphotype the basal leaves formed during and after the flowering period are undivided (basal leaves 2 and 3 sensu [76]). In both groups the above-mentioned basal leaves of the flowering period are referred to as spring leaves. These taxonomically most informative spring basal leaves (comp. [53]) were digitized from the above individuals employed for molecular investigation (or their respective populations) using a book2net Kiosk scanner. Subsequently, 2D landmark data were obtained from leaf outlines. In total, data from 95 individuals were obtained for morphometric analyses (28 leaves of *R. notabilis*, 26 of the synthetic hybrids and 41 of *R. variabilis*). Fourteen fixed 2D landmarks were depicted on the leaf outline using TpsDig2 2.16 [77]. The landmarks were localized on the tips of major lobes and in the major incisions. The program TpsRelw 1.49 [78] was used to standardize the morphometric data via generalized Procrustes analysis (GPA) [79]. Prior to subsequent multivariate analyses, landmark configurations were bilaterally symmetrized according to [80] in the program PAST 2.17c [71]. The principal component analysis (PCA) of the geometric morphometric data (i.e., Relative Warps Analysis, RWA [79]) was conducted on the entire set of 95 leaves in TpsRelw 1.49 [78]. Herein we also reconstructed the hypothetical shape of mean landmark configuration within each group. Phenetic distances among the three compared taxa were expressed as Procrustes distances among the group mean landmark configurations (as computed in TpsSmall 1.20 [81]). The statistical significance of differences between groups was computed by two-group permutation tests (9999 permutations, Euclidean metric) using PAST 2.17c [71], based on specimen scores on the first 12 relative warps (accounting for 99.99% of the total variability in shape data).

## Results

### Inter- and intraspecific variability of ITS1-ITS2 inferred from direct sequencing

**Figure 2:**
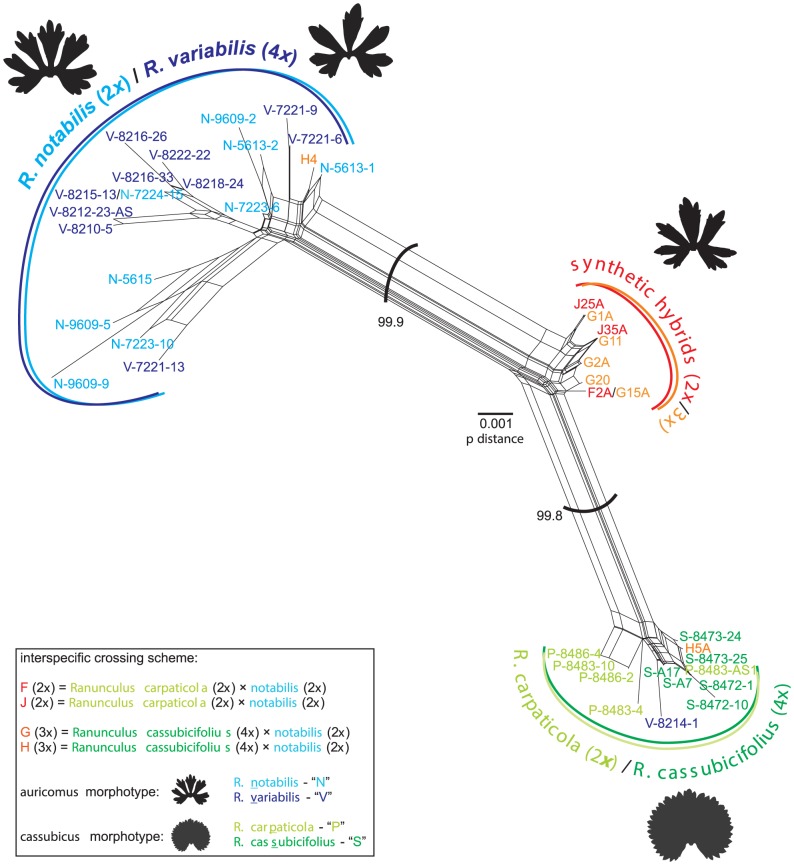
NeighborNet analysis of interspecific ITS1+ITS2 variability within the *Ranunculus auricomus* complex. NeighborNet analysis of all ITS1+ITS2 sequences obtained by direct sequencing of the studied individuals. The spring leaf silhouettes illustrate the main phenotypic differences between the two morphotypes: the *auricomus*-morphotype (characteristic for *R. notabilis*, *R. variabilis* and the synthetic hybrid *R. cassubicifolius* × *notabilis*) and the *cassubicus*–morphotype (i.e., *R. carpaticola* and *R. cassubicifolius*). Individuals belonging to *R. carpaticola* are marked as “P”, *R. cassubicifolius* as “S”, *R. notabilis* as “N” and *R. variabilis* as “V”, respectively. Identical sequences representing the same ribotype are listed in Figure 3. Bootstrap values are given for the main clusters.

**Figure 3:**
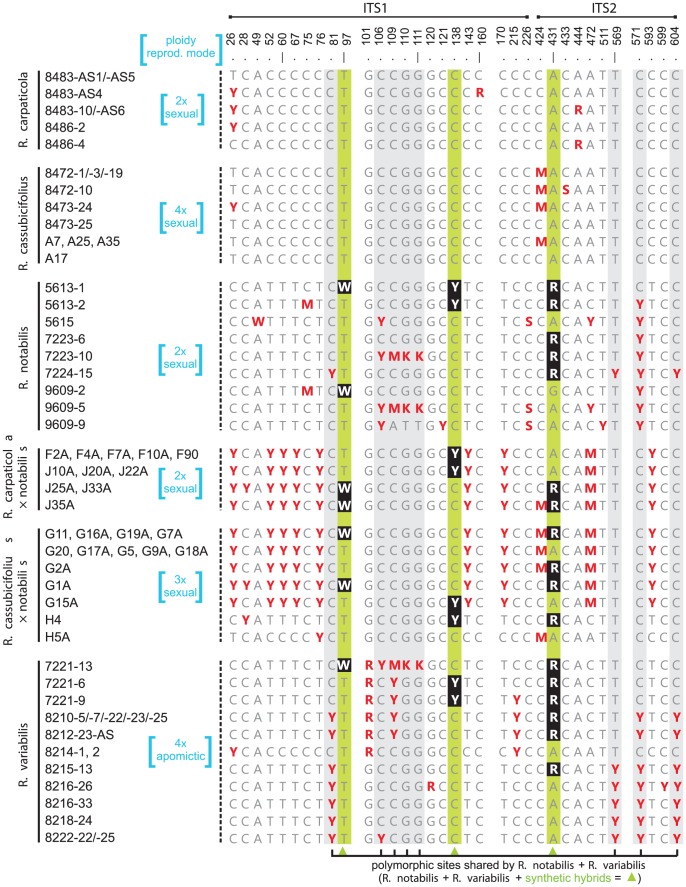
Polymorphisms of ITS1+ITS2 within the *Ranunculus auricomus* complex as detected by direct sequencing. A summary of all ITS1+ITS2 polymorphic sites (red and white letters) detected through direct sequencing. The three polymorphic sites which are shared by *R. notabilis*, its synthetic hybrid and the putative natural descendent (i.e., *R. variabilis*) are highlighted by white letters on black background and underlaid by green stripes. Those shared just by *R. notabilis* and *R. variabilis* are underlaid by grey stripes.

NeighborNet analysis of ITS sequences (Figure 2) indicated a clear genetic separation of representatives of both morphotypes, the *auricomus* (dissected basal leaves, *R. notabilis*, *R. variabilis*) and the *cassubicus* (undivided basal leaves, *R. carpaticola*, *R. cassubicifolius*). Both clusters were well supported but differed in their internal variability. The *cassubicus* cluster appeared to be more homogeneous, i.e., diploid *R. carpaticola* and tetraploid *R. cassubicifolius* were rather intermixed than well separated from each other. The genetic similarity between the *auricomus* species was slightly lower (0.995) than between the *cassubicus* species (0.997), and the mean genetic similarity between the both clades was even lower (0.982). The individuals within the *auricomus* clade exhibited a higher variability, consisting of several splits containing *R. notabilis* (diploid) intermingled with *R. variabilis* (allotetraploid). The synthetic di- and triploid hybrids generally clustered in an intermediate position between their parents, i.e., between the *auricomus* and the *cassubicus* clusters. Although originating from different maternal progenitors, all synthetic F_1_ hybrids were intermixed, forming a cluster of low internal heterogeneity (with the exception of two triploids (both from “H” population; Table 1), one of which grouped within the *auricomus*, another in the *cassubicus* clade). In total, out of 501 nucleotide positions, 34 were polymorphic in the directly sequenced ITS1-ITS2 accessions (Figure 3). All 14 polymorphic sites detected in the ITS1-ITS2 of experimental hybrids were additive, either presenting a combination of maternal (*R. carpaticola* or *R. cassubicifolius*) and paternal (*R. notabilis*) contributions (e.g., sites 52, 60 and 67), or identical to polymorphisms already existing in parental genomes (e.g., sites 26 and 97). The putative ancient hybrid *Ranunculus variabilis* displayed 15 polymorphic sites, three of which were shared with both di- and triploid synthetic hybrids. Interestingly, the three sexual species exhibited intraspecific polymorphisms, especially *R. notabilis*, which showed nine polymorphisms shared with *R. variabilis*.

### Inter- and intraindividual variability of ITS1-ITS2 inferred from cloning

**Figure 4:**
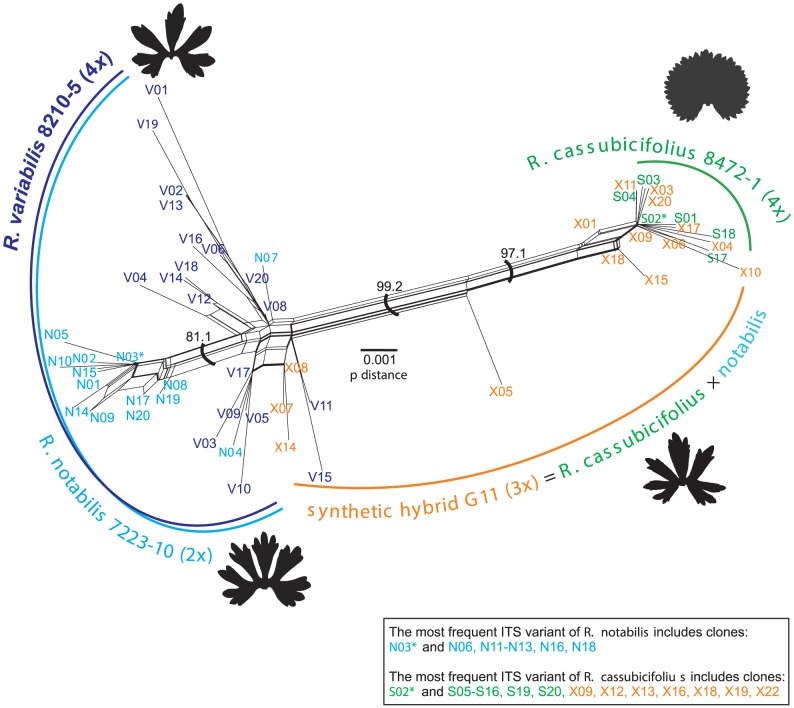
NeighborNet analysis of cloned ITS1+ITS2 variants. Particular clones belong to: *Ranunculus cassubicifolius* (“S”-clones), *R. notabilis* (“N”-clones), their synthetic hybrid (“X”-clones) and the putative hybrid, *R. variabilis* (“V”-clones). The spring leaf silhouettes illustrate the main phenotypic difference between the *auricomus* and the *cassubicus* morphotypes. Italic letters mark clones which exhibit non-compensatory base changes in the ITS1 or ITS2 secondary structures. Bootstrap values are given for the main clusters.

**Figure 5:**
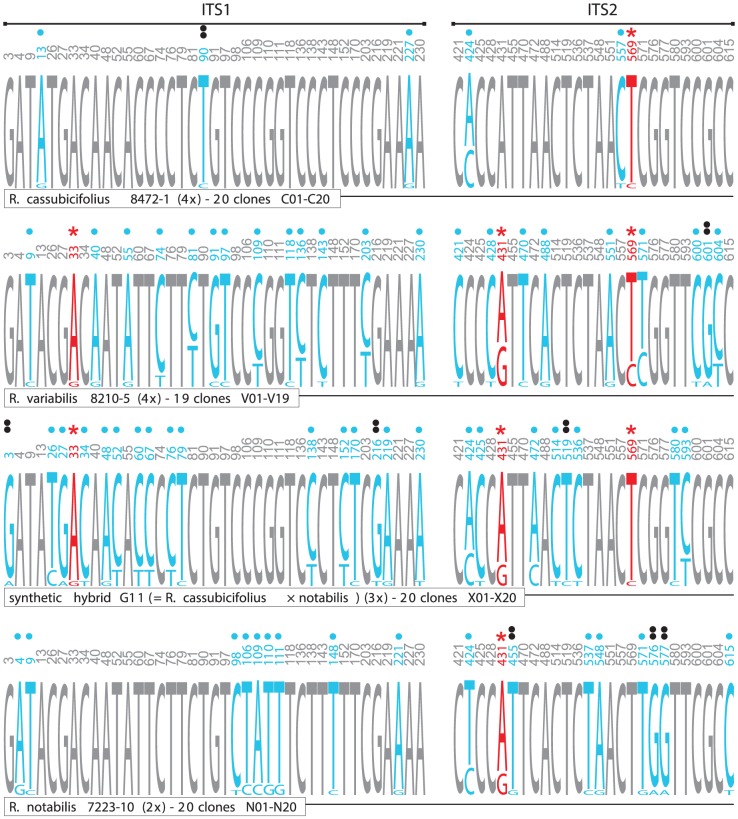
Distribution of the ITS1+ITS2 nucleotide polymorphisms within the clone libraries. The polymorphic sites are represented by blue letters and marked with dots. Red letters marked with red asterisks represent polymorphic sites, which are shared by the synthetic hybrid of *R. cassubicifolius* and *R. notabilis*, their putative natural hybrid *R. variabilis* and any of the parental species. Double black dots mark polymorphisms which occur exclusively in single clones exhibiting non-compensatory base changes in the ITS1 or ITS2 secondary structures.

**Figure 6:**
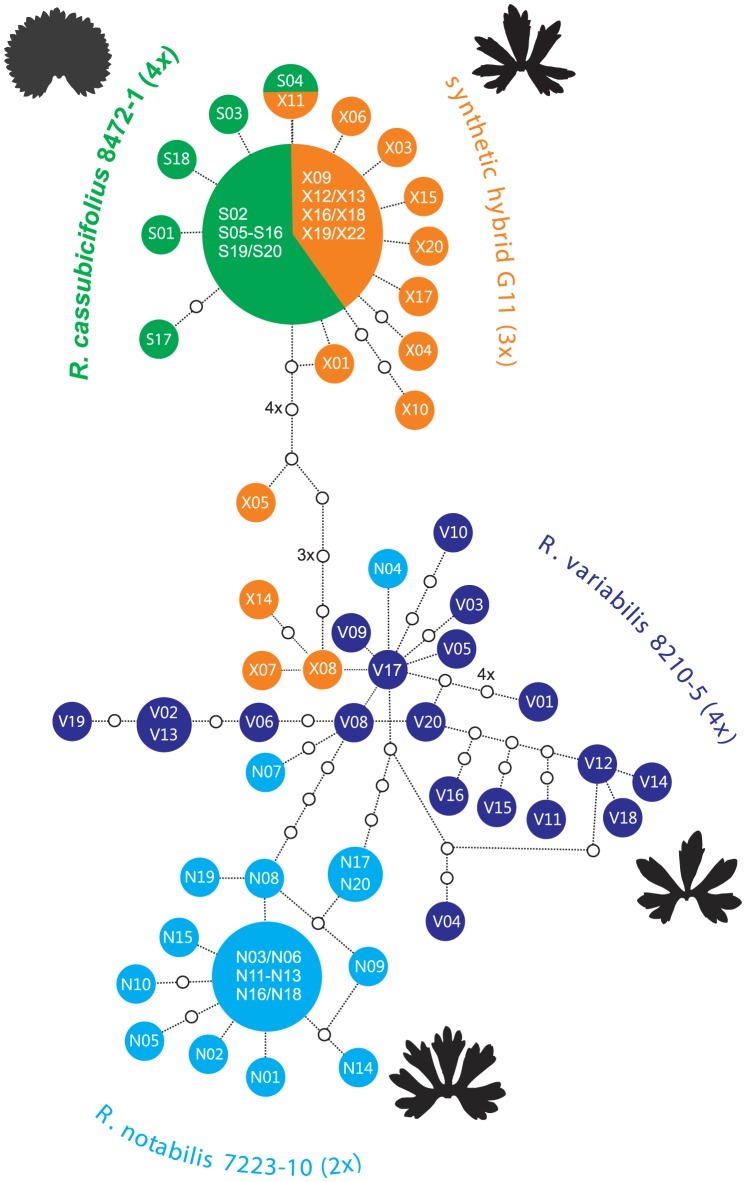
Ribotype network representing all cloned ITS1+ITS2 variants. Particular clones belong to: *R. cassubicifolius* (“S”-clones), *R. notabilis* (“N”-clones), *R. cassubicifolius* × *notabilis* synthetic hybrid (“X”-clones) and *R. variabilis* (“V”-clones). The spring leaf silhouettes illustrate the main phenotypic difference between the *auricomus and cassubicus* morphotypes. Italic letters mark clones which exhibit non-compensatory base changes in the ITS1 or ITS2 secondary structures.

The average genetic identities within clone libraries ( =  individuals) differed only slightly among the four cloned individuals and ranged from 0.998 (*R. cassubicifolius* 8472-1) to 0.996 (*R. notabilis* 7223-10). Averaged genetic similarities between the cloned individuals were highest between *R. cassubicifolius* and *R. cassubicifolius* × *notabilis* (0.999), and lowest between *R. cassubicifolius* and *R. notabilis* (0.973). Intraindividual ITS1-ITS2 variability and interindividual overlaps were visualized with NeighborNet (Figure 4). The splits diagram pointed out a high degree of intraindividual variability especially in the case of the apomict *R. variabilis*. The majority of *R. variabilis* clones clustered apart from a group of *R. notabilis* clones. However, several *R. variabilis* clones clustered in close vicinity to *R. notabilis* and even *R. cassubicifolius* × *notabilis* clones, suggesting the existence of similar ribotypes distributed over all three individuals. On the other hand, the *R. cassubicifolius* clones clustered apart from the *R. notabilis*/*R. variabilis* cluster. They formed one cluster together with the vast majority of the *R. cassubicifolius* × *notabilis* clones. As suggested from the splits graph (Figure 4), the most polymorphic individuals were the hybrids, i.e., *R. variabilis* and *R. cassubicifolius* × *notabilis*. Cloned ITS1-ITS2 amplicons from the four plant individuals exhibited 65 sites with nucleotide polymorphisms (i.e., 39 in ITS1 and 26 in ITS2, Figure 5), of which 44 were not detected by direct sequencing. Within the entire ITS1-ITS2 region the synthetic and natural hybrid individuals shared three nucleotide polymorphisms (site 33, 431, 569, as marked with an asterisk in Figure 5). Furthermore, other shared nucleotide polymorphisms were detected for *R. notabilis*, its hybrid and *R. variabilis* (site 431) and for *R. cassubicifolius*, its hybrid and *R. variabilis* (site 569) (Figure 5). The ribotype network (Figure 6) points out a remarkable overlap among the 4*x R. cassubicifolius* ribotypes (“S”-clones) and the ribotypes of its hybrid progeny *R. cassubicifolius* × *notabilis* (“X”-clones). Another prominent feature is the high amount of different ITS1-ITS2 ribotypes of *R. variabilis* (“V”-clones), where 95% of sequenced clones belonged to different ribotypes. None of these numerous ribotypes was completely identical to those from either *R. notabilis* (“N”-clones) or *R. cassubicifolius* × *notabilis* (“X”-clones).

### Phenotypic variability within the “*auricomus*” morphotype

**Figure 7:**
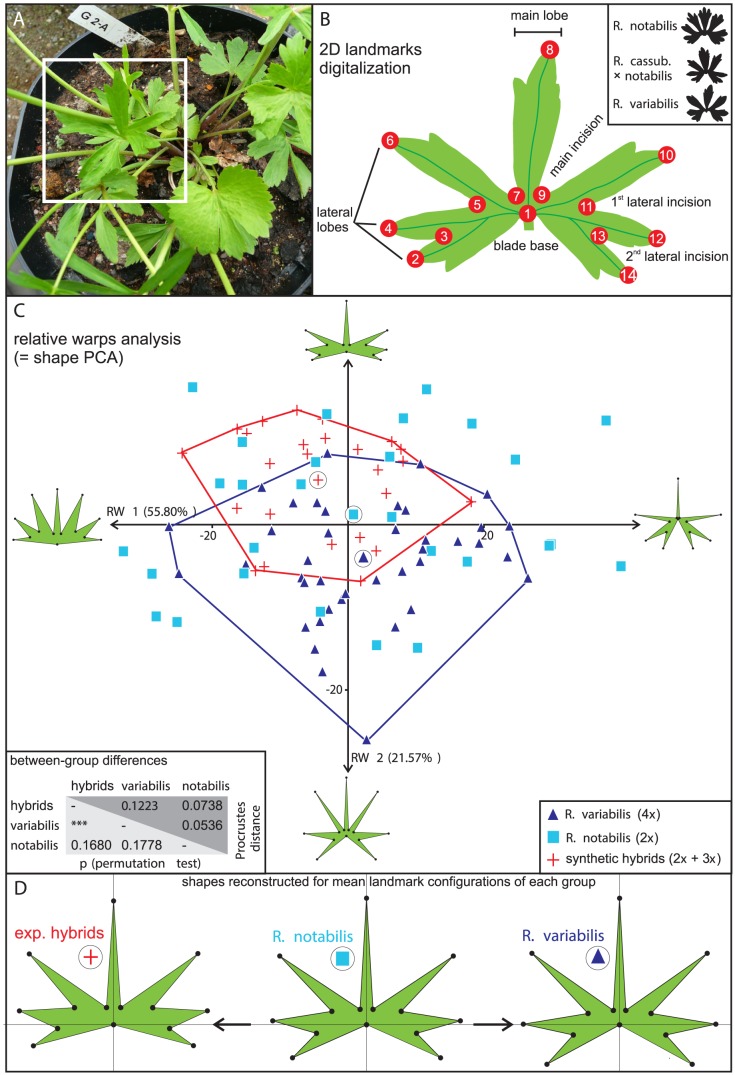
Geometric morphometric analysis of individuals exhibiting the characteristic “*auricomus*” morphology. (A) An example of the sampled fresh plant material for DNA sequencing and geometric morphometric analyses. (B) 2D landmarks digitalization on the leaf outline. (C) Principal components analysis of the shape variables (i.e., Relative warps analysis, RWA) extracted from *R. notabilis* (blue squares), *R. cassubicifolius* × *notabilis* (red crosses) and *R. variabilis* (violet triangles) 2D landmark data. (D) Mean shapes of *R. notabilis*, *R. cassubicifolius* and *R. variabilis* reconstructed from each centroid (visualized as symbols surrounded by black-lined circles) of the three scatter clusters shown in (C). The between-group differences were tested by permutation tests (lower left triangle, ***  =  p<0.001) and the distances between the mean shapes are expressed as Procrustes distances (upper right triangle).

Both di- and triploid synthetic hybrids (Figure 7a) resembled their (same) paternal parent, *R. notabilis*, having deeply dissected basal leaves (*auricomus* type). In contrast, both maternal parents (i.e., *R. carpaticola*, *R. cassubicifolius*) exhibited typical *cassubicus* morphology, characterized by non-dissected basal leaves (Figure 2). The 2D landmark data (Figure 7b) revealed slight differences between *R. notabilis*, the synthetic hybrids and *R. variabilis*. The resulting scatter plot (Figure 7c) summarizes two most prominent morphological trends within the dataset of 95 leaves. The main morphological trend (1^st^ relative warp, RW1) describes variance at the leaf basis and accounts for 55.80% of the total morphological variability. This major morphological trend appears to reflect leaf size and does not separate the three groups. Only the second morphological trend (2^nd^ relative warp, RW2), describing a gradient from robust coarsely dissected to finely dissected forms, slightly differentiated *R. variabilis* and the synthetic hybrids, assigning *R. notabilis* an intermediate position in between. The mean phenetic distance (quantified as Procrustes distance between two group centroids) was nearly the same between *R. notabilis* and *R. variabilis* (0.0536) as between *R. notabilis* and its synthetic hybrids (0.0738). The distance between *R. variabilis* and the synthetic hybrids accounted for nearly as twice as much (0.1223). This was the sole case for which the mean morphological difference (based on the scores on 12 relative warps) was statistically significant. Figure 7d visualizes the differences among the mean shapes of *R. variabilis*, *R. notabilis* and the experimental hybrids, illustrating the variation in the angle between the upper and lower part of the basal lateral lobes.

### ITS1-5.8S-ITS2 polymorphisms in the secondary structure models

**Figure 8:**
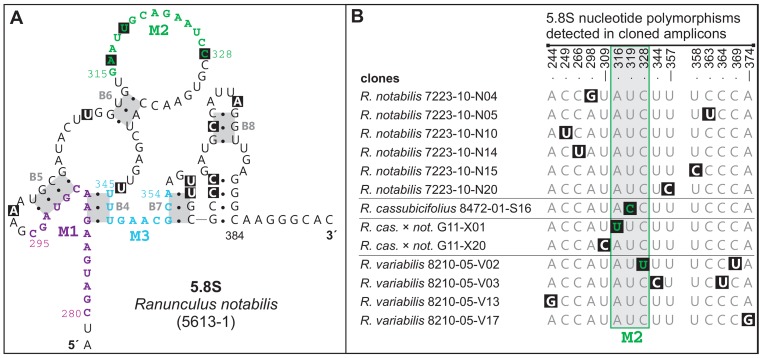
5.8S secondary structure model for *Ranunculus notabilis* 5613-1. (A) The partial 5.8S secondary structure model comprising conserved sequence regions (M1-M3), conserved helices (B5-B8) and highlighted sites affected by nucleotide substitutions (for the whole 5.8S secondary structure see Figure S1). (B) A summary Table of 5.8S nucleotide polymorphisms detected in clones.

**Figure 9:**
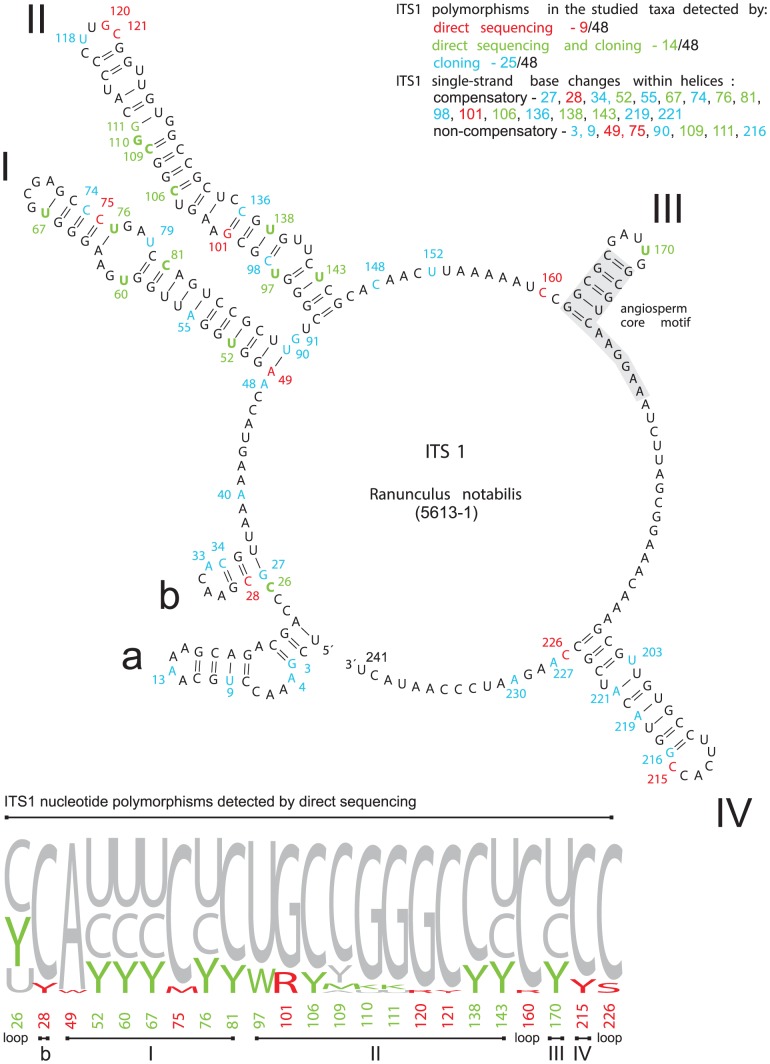
ITS1 secondary structure model for *Ranunculus notabilis* 5613-1. Polymorphic sites detected in all studied taxa were highlighted in the secondary structure model for *R. notabilis* 5613-1. The DNA weblogo summarizes all ITS1 nucleotide polymorphisms detected by direct sequencing (red letters) and both direct sequencing and cloning (green letters). Bonds within the ITS1 secondary structure are shown for all positions which can be affected by either non-compensatory or compensatory ( =  hemi-CBCs) single-stranded nucleotide polymorphisms within helices. Italic letters mark non-compensatory base changes which are present only in a single clone.

**Figure 10:**
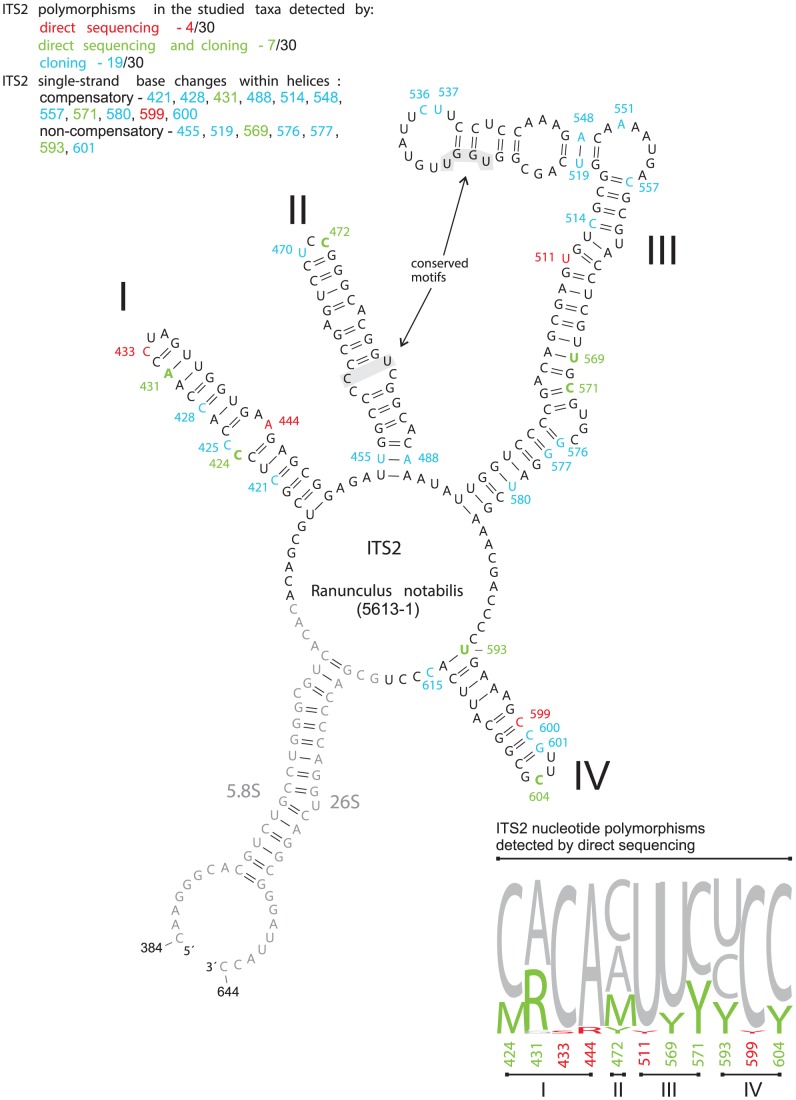
ITS2 secondary structure model for *Ranunculus notabilis* 5613-1. Polymorphic sites detected in all studied taxa were highlighted in the secondary structure model for *R. notabilis* 5613-1. The DNA weblogo summarizes all ITS2 nucleotide polymorphisms detected by direct sequencing (red letters) and both direct sequencing and cloning (green letters). Bonds within the ITS2 secondary structure are shown for all positions which can be affected by either non-compensatory or compensatory ( =  hemi-CBCs) single-stranded nucleotide polymorphisms within helices. Italic letters mark non-compensatory base changes which are present only in a single clone.

The *Ranunculus notabilis* minimum energy ITS1-5.8S-ITS2 secondary structure model (Figure S1) revealed 58% of the 241 nucleotides involved in pairings in ITS1 and 72% of the 212 nucleotides in ITS2. In all directly sequenced individuals and clones, the conserved ITS1 angiosperm motif 5′-GGCRY(4– 7n)GYGYCAAGGAA-3′ of [82] appeared as 5′- GGCGC(GAUYG)GCGUCAAGGAA-3′ in *Ranunculus* and contained no nucleotide substitutions. From the three 5.8S motifs that are also conserved across angiosperms, only one (M2) exhibited nucleotide substitutions (Figure 8), detected in three cloned amplicons. The conserved motif M3 (5′-TTTGAAYGCA-3′) as described by [40], appeared as 5′-TTTGAACGCA-3′. In order to highlight and summarize the polymorphic sites detected by direct sequencing and cloning, they were mapped onto secondary structure models of 5.8S, ITS1 and ITS2 rRNA transcripts reconstructed for *Ranunculus notabilis* 5613-1 (Figure 8a, 9, 10). In the ITS1 secondary structure we identified six hairpins (I-VI, Figure 9), in the ITS2 four hairpins (I-IV, Figure 10). Those nucleotide substitutions which affect only one strand of the double-stranded helical regions are referred to as hemi-compensatory base changes (hCBC). Considering the directly sequenced ITS1-ITS2 accessions, out of the total number of 34 polymorphic sites, 12 correspond to hemi-compensatory base changes and six base changes are not compensatory. The cloned amplicons yielded a total of 65 polymorphic sites in ITS1-ITS2. The additional DNA weblogos in Figures 9 and 10 illustrate all possible variants for each particular polymorphic site (as detected by direct sequencing), and help to recognize non-compensatory (i.e., altering the secondary structure by missing pairing within any of the helical regions) vs. compensatory polymorphisms. Within the highly conserved 5.8S region, 13 sequences exhibited polymorphisms in single clones (Figure 8b) and might therefore represent putative non-functional ITS copies or cloning/PCR artifacts.

## Discussion

Despite the tremendous popularity of the ITS nrDNA marker in angiosperm systematics, its variability on intraspecific or even on intraindividual level is still poorly known. The occurrence of ITS in multiple copies within a single genome requires more comprehensive sampling approaches to reconstruct reticulate phylogenies. Moreover, recovery of the whole range of individual variation can facilitate addressing questions regarding species evolutionary history. Here we contribute with the first detailed insight into the intraspecific ITS diversity of the *Ranunculus auricomus* species complex. Similar amount of recovered polymorphisms across both ITS1 (19%) and ITS2 (14%) regions was found in other polyploid complexes (e.g., [31], [83]).

### ITS polymorphism and morphology in the synthetic hybrids

Our investigated reproduction system consists of a single apomictic microspecies, three sexuals with different ecology/distribution areas and their synthetic hybrids. The diploid and triploid synthetic hybrids (F_1_ generation) were predominantly sexual (with a small percentage of apomictic seeds in triploids [58]) and exhibited complete additivity in the ITS1-ITS2 polymorphisms, reflecting genome contributions from their respective parents *R. carpaticola* ♀, *R. cassubicifolius* ♀ and *R. notabilis* ♂. This pattern was already observed in the NeighborNet analysis of the directly sequenced individuals, where the hybrids occupied intermediate positions between their parental species, but is also evident in shared polymorphisms in the cloned data (Figure 5). When considering all cloning data, the vast majority of cloned ribotypes of synthetic hybrids were distinctly shifted towards their maternal progenitor. This result is not surprising, as gametes of tetraploid *R. cassubicifolius* contributed two genome copies to the triploid offspring, whereas those of diploid *R. notabilis* contributed only one. Asymmetry in ITS copies in natural allopolyploidization events could have arisen from a higher number of molecular variation introduced by the tetraploid parent. Other authors [58], [84] reported on a deviation from parental additivity after hybridization, the maternal dominance being attenuated after polyploidization as well. This uneven parental genome contribution may be another reason why the putative natural hybrid, *R. variabilis*, does not match exactly the synthetic hybrids in the ribotype analysis, but appears to be more similar to *R. notabilis*. Nonetheless, the original hybridization event in nature could have happened with equal genome proportions between diploid *R. cassubicifolius*, which still occurs in the sympatric area with *R. variabilis* in one isolated population at the Austrian/Hungarian border [85]; Figure 1), and diploid *R. notabilis*. After hybrid origin, the early hybrid generations were probably sexual or only facultative apomictic, as in our experimental crosses [58]. Backcrossing of the first hybrid generations to the geographically closer parent, *R. notabilis*, or also concerted evolution of ITS copies in the first hybrid generations could have resulted in a higher genetic similarity of *R. variabilis* to *R. notabilis*.

In contrast to other studies, referring to hybrids as either intermediate phenotypes or mosaics of their parents [3], [46], [86], [87] our cloned ITS data underlined the remarkable incongruence between the genotype and leaf morphology of the *Ranunculus cassubicifolius* × *notabilis* triploid hybrid. While including two genome copies from the maternal parent, the hybrid retained the clearly distinct paternal-like leaf morphology. Noack [88] described pentaploid hybrids that were more similar to the paternal progenitor rather than to the triploids which were morphologically intermediate. Further evidence against simple intermediacy was provided in [89] on *Sorbus* hybrid microspecies, observing leaves of mosaic, intermediate or parental-like features. As already discussed by [29], [85], hybrid progeny in *R. auricomus* exhibits a continuous range of morphotypes ranging from extreme forms over intermediates to those completely identical with one of their parents. In the case of our synthetic hybrids, the observed parental-like leaf phenotype may also have an epigenetic basis. Results from our synthetic hybrids show that the parentage and evolutionary origin cannot be readily predicted from morphological data, while ITS polymorphisms reflect the genome additivity of the parental species.

### ITS polymorphism, reticulate evolution and mutation accumulation in the *R. auricomus* complex

Compared to allozyme genetic identity within *R. notabilis* (0.965) and *R. variabilis* (0.940) assessed by [55], the ITS1-ITS2 cDNA differentiation was in both cases considerably lower in these species (*R. notabilis*: 0.996, *R. variabilis*: 0.989). Although there was no particular ribotype shared between *R. notabilis* and *R. variabilis*, they clustered together. Liao et al. [90] recovered ten ITS polymorphisms in allotetraploid *R. cantoniensis*, a similar amount compared to our 13 polymorphisms detected in the allotetraploid *R. variabilis*. Intraindividual variability in ITS nrDNA can be maintained in apomictic hybrid species, as documented already by [30] in *Amelanchier*. Since apomictic reproduction generally results in seed formation without meiosis and syngamy, the homogenizing effects on ITS copies and concerted evolution are lacking. Hence, agamic lineages can preserve ITS polymorphisms from hybrid origin and can accumulate mutations independently in the polyploid genome. This so-called Meselson effect was in fact observed in hexaploid hybrids of the *R. auricomus* complex via large scale Illumina RNA sequencing [91]. Particularly in the case of allotetraploid *R. variabilis*, the observed amount of ITS polymorphisms may be indicative for lack of concerted evolution and post-origin mutation accumulation, a phenomenon frequently observed in apomictic plant polyploid species complexes [21], [30].

Other than in the case of functional ITS copies, other authors [37] found most of the non-functional and chimeric ITS copies in a few million years old clade within *Gnetum*. We detected the largest number of putative non-functional copies (which contained polymorphisms in 5.8S region) in *R. notabilis* (20% of the clones) and in *R. variabilis* (16% of the clones). However, two polymorphisms in 5.8S were detected in the synthetic hybrid as well (10% of the clones). The ancient progenitor-derivative relationship between *R. notabilis* and *R. variabilis*, as proposed already by [55] based on allozyme data, was further supported by the observed overlaps in 10 shared polymorphic sites as inferred from direct sequencing. The high intraindividual ITS polymorphism in *R. notabilis* individuals is unusual for a sexual species. Regarding the assessed split between *R. notabilis* and *R. cassubicifolius* about 0.9 Ma ago [54], such a high amount of ITS polymorphism is not expected. The most plausible explanation is that *R. variabilis* has introgressed into *R. notabilis* in early sexual hybrid generations, or also recently via backcrossing as the pollen donor, which is reasonable from the overlap of distribution areas (Figure 1) and the co-occurrence of the two taxa in some of the sites [55].

### Secondary structure, compensatory base changes and non-functional ITS copies

The amount of detected nucleotide pairings in RNA transcripts in the *Ranunculus auricomus* complex was higher compared to the observations by [65] on Asteraceae suggesting at least 20% of ITS1 and 38% of ITS2. Nevertheless, neither among the sexual species nor between them and the apomict *R. variabilis* did we recover any CBCs. The importance of these structural features for species concepts is still under debate [33], [34]. The strongly reduced fertility of our synthetic hybrids [92] suggests that their sexual parents are distinct biological species. Hence, the relevance of CBCs for delimitation of species, and for reconstructing reticulate evolution and interspecific hybridization remains unclear. However, the occurrence of the numerous compensatory single-strand nucleotide polymorphisms (i.e., hemi-CBCs) might be a hint for an ongoing process resulting in a complete CBC during further divergence of taxa. More than a half of the hemi-compensatory base changes in ITS1-ITS2 were present exclusively in the cloned amplicons. A suspected artificial nature can be excluded because the observed substitutions retain the nucleotide bonds and the secondary structures. In contrast, the majority of non-compensatory substitutions were present in single clones only and hence, may represent PCR artifacts. Furthermore, the secondary structure analysis of the 5.8S region indicated no substitutions in the directly sequenced amplicons, but numerous substitutions in single clones. Sixteen percent of the analyzed clones showed polymorphisms in the 5.8S, belonging to 15 different variants, an observation close to [41] from *Mammillaria*. In contrast to the mutations present in the DNA template, the nucleotide substitutions in otherwise conserved 5.8S might have been introduced during PCR reaction and *E. coli* cell division [93]–[95]. Nevertheless, Záveská and collaborators [21] identified in *Taraxacum* considerable polymorphisms in 5.8S (3.7%), considering all these ITS copies to be functional with regard to their secondary structures. Similarly, we neither observed structural changes within 5.8S introduced by the substitutions nor did we recover any recombinant/chimeric sequences through recombination detection algorithms or via NeighborNet analysis. Additionally, the patterns of methylation-induced substitutions or GC content variation (Figure S2) both seem to be insufficiently informative to separate between functional and non-functional/artificial ITS copies. Therefore, we suggest that the constraint of the secondary structure preservation is a useful tool for the DNA data quality assessment in cloned amplicons.

## Supporting Information

Figure S1 **ITS1-5.8S-ITS2 secondary structure model for** ***Ranunculus notabilis*** **5613-1.** The secondary structure model for *R. notabilis* 5613-1 served as a template to locate all ITS1-5.8S-ITS2 polymorphisms detected within this study.
(EPS)

Figure S2 **Scatter plot of GC content of ITS1, 5.8S and ITS2 sequence subregions.** 3D scatter plot of GC contents for all 79 sequenced clones and four directly sequenced individuals lacking ambiguous polymorphic sites. Underlined letters indicate clones that contained substitutions in the 5.8S region and italic letters mark clones which exhibited non-compensatory base changes in either ITS1 or ITS2 secondary structures.
(EPS)
